# Is the Demand for Alcoholic Beverages in Developing Countries Sensitive to Price? Evidence from China

**DOI:** 10.3390/ijerph8062124

**Published:** 2011-06-09

**Authors:** Guoqiang Tian, Feng Liu

**Affiliations:** 1 College of Economics and Management, China Agricultural University, No. 17, Qinghuadong Road, Haidian District, Beijing 100083, China; E-Mail: tiangq@cau.edu.cn; 2 School of Economics, Shanghai University of Finance and Economics, 777 Guoding Road, Shanghai 200433, China

**Keywords:** alcoholic beverage, drinking, alcohol demand, price elasticity, beer, liquor

## Abstract

Economic literature in developed countries suggests that demand for alcoholic beverages is sensitive to price, with an estimated price elasticity ranging from −0.38 for beer and −0.7 for liquor. However, few studies have been conducted in developing countries. We employ a large individual-level dataset in China to estimate the effects of price on alcohol demand. Using the data from China Health and Nutrition Survey for the years 1993, 1997, 2000, 2004 and 2006, we estimate two-part models of alcohol demand. Results show the price elasticity is virtually zero for beer and only −0.12 for liquor, which is far smaller than those derived from developed countries. Separate regressions by gender reveals the results are mainly driven by men. The central implication of this study is, while alcohol tax increases can raise government revenue, it alone is not an effective policy to reduce alcohol related problems in China.

## 1. Introduction

According to World Health Organization [1], alcohol consumption is declining in most of the developed countries, but rising in many of the developing countries. Drinking alcohol is associated with a number of adverse health effects and social problems, such as oesophageal cancer, epileptic seizures, liver cirrhosis, chronic pancreatitis, road injuries, domestic violence, homicide, household poverty, and so on. The global burden of alcohol use over all countries was estimated to be 4% of the total disability adjusted life years lost, which makes it more damaging than tobacco (2.6%). Alcohol causes 1.8 million annual deaths (3.2% of total) worldwide, with 80% of this excess mortality occurring in the developing regions of the world [2].

To reduce alcohol-related problems, governments and communities have made enormous efforts that have included health promotion programs, counter-marketing, increasing alcohol taxes, controls over physical availability, regulation of product labels, and limits on advertising, *etc.* Among these, the most effective policy is probably the alcohol taxation [3]. Economics literatures show that consumers drink less when alcoholic beverage prices are increased [4]. While a large body of empirical literature related to alcohol demand exists, virtually all of this work has focused on developed nations. In a meta-analysis of own-price elasticity of alcoholic beverages, Fogarty [5] reviewed 64 studies for 18 countries. Only one of them was for a developing country. The average price elasticity in developed countries is −0.38 for beer, −0.77 for wine, and −0.7 for liquor [5].

The study of alcohol consumption among residents of a developing economy may be of particular interest because, as others have argued, the lower levels of both income and education may make the average consumers in those countries more sensitive to changes in prices and taxes than the average consumers in economies with higher levels of income and education. Taking cigarettes (which are similar to alcohol in terms of addition and harm to health) as an example, an early review study [6] suggested the price elasticity of cigarettes in developing countries is higher in absolute value than that in developed countries. However, more recent studies of smoking in China and Russia [7,8] have cast doubts on this conclusion. The sparse demand studies on alcohol conducted in China generally find significantly larger price effects than those in developed countries. Pan *et al*. [9] employ an urban household survey from three cities (Beijing, Tianjin, Shanghai) and one province (Jiangsu) and find the price elasticity ranges from −1.36 for wine and −0.9 for beer. Yu and Abler [10] use provincial level data of ten years for rural areas and find the overall alcohol price elasticity at −1.53. However, both studies have no direct measure of alcohol prices. They compute alcohol prices as expenditures on alcohol divided by the total quantity of alcohol consumed. Obviously this calculation is subject to endogeneity bias because its denominator is the endogenously determined quantity. Other limitations include small sample size in Pan *et al*. [9] and aggregate data in Yu and Abler [10]. The contribution of our study is twofold. First, we have direct measure of alcohol prices and we explore three different models to address possible price endogeneity concerns. Second, we use large individual level data of multiple years to permits more extensive examination of variation in price sensitivity across demographic subgroups.

## 2. Data and Methods

Our data come from the China Health and Nutrition Survey (CHNS)—a panel survey which began in 1989 with a sample of about 4400 households with a total of 16,000 individuals. Follow-up surveys were administered in 1991, 1993, 1997, 2000, 2004 and 2006. The CHNS has a multi-stage random cluster design and surveys people from nine provinces in China (Liaoning, Heilongjiang, Jiangsu, Shandong, Henan, Hubei, Hunan, Guangxi, and Guizhou). These nine provinces are quite diverse in terms of their economic development and geographic features and are selected to capture a wide range of socioeconomic and urban–rural characteristics in China. The CHNS also includes a community survey, which in CHNS refers to the respondent’s neighborhood in urban areas and to the village or town in rural areas. The community heads or appropriate vendors/salespersons were asked to report the prices of most commonly used commodities, including alcoholic beverages. Wine prices were not reported, so we only include beer and liquor in this paper.

Our study makes use of questions on drinking behavior asked in wave 1993, 1997, 2000, 2004 and 2006 of the survey. These questions ask respondents to report whether or not they drank any alcoholic beverages in the past year, the frequency of drinking, and, for each type of alcohol (beer, wine, and liquor), how much they drink each week. In this study, we have a pooled sample of 44,025 individuals.

We estimate two-part models [11,12] of alcohol demand, which is appropriate because a zero value of our dependent variable represents a genuine choice of not drinking alcohol; *i.e.*, it is not due to nonresponses:

(1) $$Y_{\text{ijt}}=\beta_{0}+\beta_{1}\times {\text{Pr}\text{ice}}_{\text{jt}}+\beta_{2}X_{ijt}+\beta_{3}T_{t}+{ɛ}_{ijt}$$

The subscript *i* refers to individuals, *j* to communities, and *t* to years. The first part of the model is a biprobit (considering decision to drink beer and decision to drink liquor are correlated) in which Y = 1 if the respondent reports drinking any alcohol. The second part of the model is an ordinary least squares (OLS) regression in which Y is the log of the amount of alcohol consumed each week, conditional on drinking. The regressor of interest is the price of alcohol beverage at the community-level. X is a vector of individual characteristics, including sex, age, minority status, household size, per capita household income, education, marital status, employment status, and occupation. T is a set of year fixed effects. Year fixed effects allow us to control for any fixed year-specific characteristics that are correlated with both alcohol prices and alcohol consumption. For the purpose of identifying the price effect on alcohol consumption, we should be cautious about one potential problem with Equation (1). Price could be endogenous because prices may be correlated with other community-level characteristics that also affect drinking behavior. Following Lance *et al*. [7], we estimate three different models to address the concerns of price endogeneity problem: (1) Simple pooled cross-sectional data; (2) Province fixed effects model: In this model we add controls for province dummies for nine provinces; (3) City/county fixed effects model: In this model we include dummy variables for each city or county. With fixed effects models, we can control confounding unobserved community-level characteristics. For all models, we run separate regressions for beer and liquor. We also employ robust standard errors to allow for clustering at the community level.

## 3. Results and Discussion

**Figure 1.**
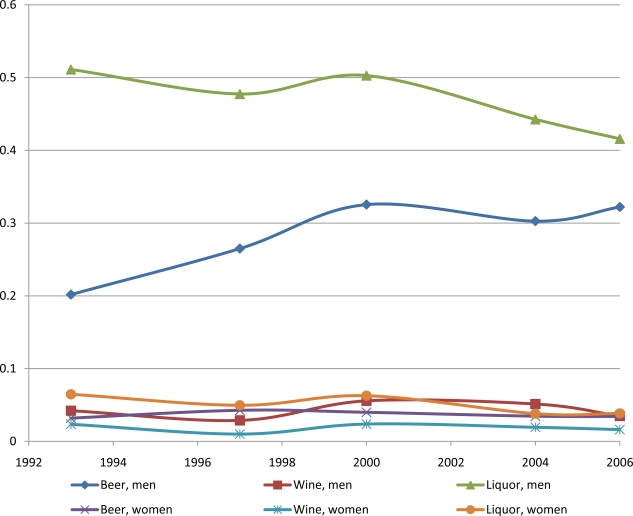
Probability of alcohol consumption by sex and by the type of alcohol.

Figure 1 displays the trend of probability of alcohol consumption by sex and by the type of alcohol. Clearly very few women drink alcohol. For men, wine consumption is also scarce (less than 5%). This is one possible reason that wine price is not reported in the CHNS survey. The probability of liquor consumption is declining over time, from over 50% in 1993 to 41% in 2006. In contrast, the probability of beer consumption is rising more than 50%, from only 20% in 1993 to 32% in 2006. Table 1 presents basic descriptive statistics separately by sex. It shows substantially more men drink in China than women. For beer drinking, the prevalence rate is 29% for men and only 4% for women; for liquor drinking, it is 47% for men and 5% for women. In addition, among alcohol drinkers, men consume about 55% more beer per week than do women (3.67 *versus* 2.11 bottles per week). In our sample, women were far more likely than men to have no formal education (37% *versus* 21%) and were less likely having completed schooling past primary school. Men and women are distributed equally across the different geographies (urban and rural areas) and across waves of the survey.

For the independent variables, besides those in Table 1, we also control for employment status (seven categories: employed, seeking work, house-worker, disabled, student, retired, not working for other reasons) and occupation (seven categories: professional worker, general office staff, administrator/executive/manager, farmer, skilled or service worker, non-skilled worker, others). Estimation results for overall sample are presented in Table 2. For beer, the price has a negative and statistically significant impact on both drinking participation and conditional alcohol demand in the baseline specification. However, the significance disappears with the introduction of province or city/county level fixed effects. Fixed effects models are preferred because they take into account the confounding unobserved community-level characteristics. For liquor, the price effect is generally significant in all specifications. But the estimates are quite small. The participation elasticity ranges from −0.007 to −0.017 and the intensity elasticity is around −0.1 consistently across three specifications. Based on the specification of city/county level fixed effects, the total (participation+ intensity) price elasticity is −0.12, which is far smaller in absolute value than those recovered in the earlier Chinese studies [9,10]. The main reason is that earlier studies have no direct measure of prices of alcoholic beverages.

At least two factors might explain such a low price effect in China. First, Chinese cultural norms encourage social drinking. Especially among businessmen, alcohol drinking is seen as a necessary vehicle for success. Alcohol drinking is also believed to help maintain good relations between supervisors and employees and among colleagues [13]. Therefore, many alcoholic beverages are consumed at public expense. If people do not use their own money to pay alcoholic beverages, they are not sensitive to the price. Of course, social drinking is not universal among all developing countries. Thus, this particular reason cannot be generalized to other developing nations. Second, in developed countries, alcohol tax increase are often accompanied by other alcohol control policies, such as restricting access to youth, reduce sales to intoxicated patrons, restrict alcohol advertising, policy targeted drunk-driving, *etc.* These policies are likely to both confound and interact with the price effects. Since many of these are not currently present in many developing nations such as China, this could result in lower price responsiveness.

It is important to note the difference between beer and liquor. Liquors usually have an alcoholic content of 50% to 60%. Given the same amount of alcoholic beverages, liquor is more harmful than beer. Our results show price elasticity for liquor is bigger than that for beer. Therefore, in order to reduce alcohol consumption and alcohol-related problems, it might be more effective to implement different tax rates for beer and liquor.

We further run separate regression for men and women. The results are presented in Table 3 for men and Table 4 for women. The results for men are very similar to that for overall sample. For women, the price effect is either too small or not statistically significant at all.

## 4. Conclusions

This paper finds a very small price elasticity of alcoholic beverage consumption in China (virtually zero for beer and −0.12 for liquor), in contrast to those derived from developed countries and those from earlier work on developing countries. Separate regressions by gender reveals the results are mainly driven by men. The central implication of this study is that while alcohol tax increase can raise government revenue, it alone is not an effective tool to reduce alcohol-related problems in China.

## Figures and Tables

**Table 1.** Sample statistics.

**Variables**	**Overall**	**Men**	**Women**
Probability of beer consumption	16%	29%	4%
Amount of beer per week, among the consuming (bottles)	3.51 (5.06)	3.67 (5.09)	2.28 (4.67)
Probability of liquor consumption	25%	47%	5%
Amount of liquor per week, among the consuming (50 g)	10.86 (13.37)	11.49 (13.75)	5.23 (7.31)
Price of beer (bottle/640 mL)	2.39	2.40	2.39
Price of liquor (500 g)	5.35	5.34	5.37
Marital status			
- Never married (reference)	0.13	0.16	0.10
- Married	0.79	0.80	0.79
- Widowed or divorced	0.08	0.04	0.11
Education			
- Less than primary school (reference)	0.29	0.21	0.37
- Primary school	0.19	0.20	0.18
- Middle school	0.30	0.35	0.26
- High school	0.16	0.19	0.14
- College or higher	0.05	0.06	0.04
Age			
- 18–24 (reference)	0.10	0.11	0.09
- 25–40	0.32	0.31	0.32
- 41–59	0.34	0.39	0.31
- 60 or older	0.24	0.19	0.28
Minority	0.12	0.12	0.12
Household income (in 1000 RMB, inflated to 2006)	21.67 (25.87)	21.83 (25.62)	21.52 (26.11)
Household size	3.94 (1.54)	3.94 (1.52)	3.95 (1.55)
Smoking	0.30	0.56	0.04
Rural (=1 if in rural area, 0 otherwise)	0.65	0.65	0.65
Year			
- 1993 (reference)	0.17	0.17	0.17
- 1997	0.21	0.22	0.21
- 2000	0.20	0.20	0.20
- 2004	0.21	0.21	0.21
- 2006	0.21	0.21	0.21
Sample size	44025	21398	22627

* Standard deviation for continuous variables in parentheses.

**Table 2.** Price elasticities of alcohol demand in China, overall sample.

	**Pooled**	**Province Fixed Effects**	**City/county Fixed Effects**
**Beer**			
- Probability	−0.057 *** (0.011)	0.019 (0.012)	0.009 (0.011)
- Conditional level	−0.292 *** (0.059)	−0.036 (0.056)	−0.055 (0.058)
**Liquor**			
- Probability	−0.007 (0.005)	−0.012 ** (0.006)	−0.017 *** (0.005)
- Conditional level	−0.102 *** (0.027)	−0.103 *** (0.027)	−0.101 *** (0.026)

Robust standard errors in parentheses. Statistical significance (based on a two-tailed test) is indicated with asterisks:

*** P < 0.01,

** P < 0.05,

* P < 0.1.

**Table 3.** Price elasticities of alcohol demand in China, Men.

	**Pooled**	**Province Fixed Effects**	**City/county Fixed Effects**
**Beer**			
- Probability	−0.106 *** (0.022)	0.035 (0.024)	0.020 (0.023)
- Conditional level	−0.316 *** (0.062)	−0.055 (0.060)	−0.073 (0.061)
**Liquor**			
- Probability	−0.007 (0.009)	−0.019 * (0.011)	−0.027 *** (0.009)
- Conditional level	−0.111 *** (0.028)	−0.113 *** (0.028)	−0.112 *** (0.027)

Robust standard errors in parentheses. Statistical significance (based on a two-tailed test) is indicated with asterisks:

*** P < 0.01,

** P < 0.05,

* P < 0.1.

**Table 4.** Price elasticities of alcohol demand in China, Women.

	**Pooled**	**Province Fixed Effects**	**City/county Fixed Effects**
**Beer**			
- Probability	−0.023 *** (0.006)	0.008 (0.006)	0.002 (0.006)
- Conditional level	−0.090 (0.105)	0.107 (0.141)	0.054 (0.145)
**Liquor**			
- Probability	−0.005 * (0.003)	−0.003 (0.003)	−0.005 ** (0.003)
- Conditional level	−0.023 (0.043)	−0.029 (0.048)	0.010 (0.049)

Robust standard errors in parentheses. Statistical significance (based on a two-tailed test) is indicated with asterisks:

*** P < 0.01,

** P < 0.05,

* P < 0.1.
